# Trace amine-associated receptors as potential targets for the treatment of anxiety and depression

**DOI:** 10.3389/fphar.2025.1598048

**Published:** 2025-04-25

**Authors:** Zelong Li, Luoting Wan, Jing Dong, Jinquan Li, Jianfeng Liu

**Affiliations:** ^1^ School of Medicine, Wuhan University of Science and Technology, Wuhan, Hubei, China; ^2^ College of Life Sciences and Health, Wuhan University of Science and Technology, Wuhan, Hubei, China

**Keywords:** TAAR, stress, depression, trace amine, anxiety

## Abstract

In the metabolic pathways associated with major biogenic amines, such as dopamine, noradrenaline, and serotonin, there exists a group of compounds known as trace amines. These trace amines share structural similarities with the major biogenic amines. Since the discovery of trace amine-associated receptors (TAARs) that are activated by trace amines, numerous studies have suggested that these receptors, particularly the TAAR1 subfamily, play a role in modulating the stress response and are involved in stress-related psychiatric disorders, including depression, bipolar disorder, and anxiety. Research indicates that TAAR1 regulates the release of neurotransmitters like dopamine and serotonin, which may be a potential mechanism underlying the involvement of trace amines and TAAR1 in response to stress. Several selective TAAR1 agonists have been evaluated in various animal models of depression and anxiety, showing that these compounds can be effective in alleviating depressive and anxiety-like behaviors. Additionally, TAAR5 has also been found to have an effect on anxiety; it is proposed that a TAAR5 antagonist might produce anxiolytic effects. Despite our limited understanding of the underlying mechanisms through which TAARs regulates stress-related disorders, current evidence strongly suggests that TAAR ligands could represent novel pharmacotherapy for treating psychiatric disorders such as depression, bipolar disorder, and anxiety disorders like post-traumatic stress disorder (PTSD). This offers hope for more effective and safer treatment options in the field of mental health.

## Introduction

In the intricate metabolic pathways governing major biogenic amines like dopamine (DA), noradrenaline (NE), and serotonin (5-HT), there exists a group of compounds known as trace amines (Zucchi et al., 2006; Gainetdinov et al., 2018). These trace amines share structural similarities with the major biogenic amines and typically include β-phenylethylamine (β-PEA), p-octopamine (p-OA), m-octopamine (m-OA), tryptamine (TRP), and both p-tyramine (p-TYR) and m-tyramine (m-TYR) (Zucchi et al., 2006). Historically, extensive research had been dedicated to unraveling the diverse functions of trace amines, paralleling efforts directed toward understanding major amines. In invertebrates, trace amines have been shown to play pivotal roles, often functioning as principal neurotransmitters in various species (McClung and Hirsh, 1999; Evans et al., 1976). However, their roles in vertebrates remain shrouded in mystery, primarily due to the significantly lower concentrations of trace amines compared to major amines found in the mammalian brain (Grandy, 2007). Despite this disparity, it is important to recognize that trace amines may possess vital physiological influences (Grandy, 2007; Nguyen and Juorio, 1989; David et al., 1984). Early studies utilizing various neuropharmacological, behavioral pharmacological, and electrophysiological techniques have illuminated the profound capability of trace amines to modulate the release and function of DA, NE, and 5-HT, thereby influencing a broad spectrum of behaviors associated with these neurotransmitters (Zucchi et al., 2006; Liu et al., 2024). While initial hypotheses posited trace amines as potential neurotransmitters in their own right, subsequent research revealed that identifying their specific receptors has proven elusive. Instead, these compounds have been characterized as “false neurotransmitters,” a term that describes substances mimicking the actions of traditional neurotransmitters within the nervous system.

In 2001, two independent research teams made significant progress by successfully cloning a new group of G-protein-coupled receptors (GPCRs) that can be activated by trace amines (Bunzow et al., 2001; Borowsky et al., 2001). This accomplishment was achieved through a polymerase chain reaction approach using primers derived from the sequences of 5-HT receptors or catecholamine receptor gene family (Bunzow et al., 2001; Borowsky et al., 2001). Although there were proposals to name these trace amine receptors in accordance with the convention of labeling receptors after their endogenous agonists (Maguire et al., 2009), they have more commonly been referred to as trace amine-associated receptors (TAARs) in the scientific literature. For clarity and consistency, we will adopt the terminology of TAAR in this review. Further research has identified a total of nine distinct families of TAARs present in the mammalian brain (TAAR1-9). Among these families, the TAAR1 subfamily stands out as the most thoroughly characterized, especially concerning its physiological roles and associations with various brain disorders. Although TAAR1 is expressed at lower levels, it has been found in several critical brain regions, including the ventral tegmental area (VTA), striatum, substantia nigra, prefrontal cortex, amygdala, basal ganglia, and hypothalamus (Bunzow et al., 2001; Borowsky et al., 2001). Notably, electrophysiological studies have revealed that TAAR1 has the ability to negatively modulate the activity of DA and 5-HT transmission, suggesting its potential regulatory role in the nervous system.

Recent preclinical investigations have indicated that TAAR1 may play a pivotal role in a variety of psychiatric disorders, including schizophrenia, sleep disturbances, drug addiction, and stress-related disorders such as depression, bipolar disorder, and anxiety (Gainetdinov et al., 2018; Liu et al., 2024; Rantala et al., 2021; Maercker et al., 2022; Berry et al., 2017; Liu et al., 2022; Revel et al., 2011; Revel et al., 2013; Black et al., 2017; Goonawardena et al., 2019). In this review, we will explore recent advancements in our understanding of TAAR1’s role in stress response and related disorders, as well as the promising potential of TAAR1 agonists for treating these disorders. We aim for this review to illuminate the significant role that the trace amine system and TAARs play in the etiology of stress-related disorders.

## Trace amines in stress response and stress-related disorders

Early studies have linked trace amines to the stress response, highlighting alterations in various trace amines among patients with major depressive disorder (MDD) (Davis and Boulton, 1994; Sabelli and Javaid, 1995). In an animal study, β-phenylethylamine (β-PEA) administration induced the release of corticotrophin releasing hormone (CRH), increased CRH mRNA expression, and promoted the plasma levels of adreno-corticotrophin hormone (ACTH) and corticosterone in responses to stress (Kosa et al., 2000). These results suggest that β-PEA could regulate the hypothalamic-pituitary-adrenal (HPA), a system of glands and hormones that have been extensively implicated in stress response (Kosa et al., 2000). It was reported that human subjects after their initial experience of parachuting exhibited high levels of urinary β-PEA, providing further evidence of its role in regulating stress response (Paulos and Tessel, 1982). β-PEA is mainly produced from phenylalanine by the enzyme aromatic L-amino acid decarboxylase via enzymatic decarboxylation and metabolized by monoamine oxidase B. Thus, an increase in urinary β-PEA levels may be attributed to a stress-induced surge in phenylalanine or a reduction in MAO-B activity (Paulos and Tessel, 1982).

There is evidence that deficient PEA expression might be related to MDD. In comparison to healthy subjects, individuals with MDD showed lower levels of urinary phenylacetyl aspartate (PAA), which is the primary metabolite of β-PEA in the brain (Sabelli et al., 1983). Notably, the PAA excretion levels in untreated patients were found to be similar to those in patients undergoing antidepressant treatment (Sabelli et al., 1983). These findings point to a compelling association between low β-PEA level and MDD. In alignment with the dysregulated expression of β-PEA in patients with MDD, it has been observed that β-PEA administration produces sustained antidepressant effects in patients with MDD or bipolar disorder, including ones have not responded to conventional treatments (Sabelli et al., 1996). While these studies suggest a potential link between β-PEA and MDD, there are others that challenge this view. For instance, one study revealed that while metabolites of tyramine and octopamine were found to be deficient in patients with depression, significant alterations in β-PEA levels were not observed (Sandler et al., 1979). Moreover, the previous findings of decreased PAA excretion may not accurately reflect PEA excretion. A study demonstrated that urinary β-PEA levels did not correlate with PAA excretion (DeLisi et al., 1984), and moreover, β-PEA level was not associated with depressive symptoms of patients with depression (DeLisi et al., 1984). Nevertheless, a more recent study revealed that β-PEA prevented chronic corticosterone-induced depressive-like behaviors and reduction in BDNF-dependent signaling pathways in the hippocampus (Lee et al., 2020). A recent study found that acute stress can activate neurons known as D-neurons in the lateral habenula, which are involved in synthesizing and releasing trace amines (Yang et al., 2023). Importantly, intervening in the activity of D-neurons can significantly influence depressive-like behaviors through a neuronal circuit connecting the lateral habenula, rostromedial tegmental nucleus, and ventral tegmental area (Yang et al., 2023). In summary, current research on the relationship between trace amines and depression yields mixed results and requires further investigation to draw a conclusion.

**FIGURE 1:**
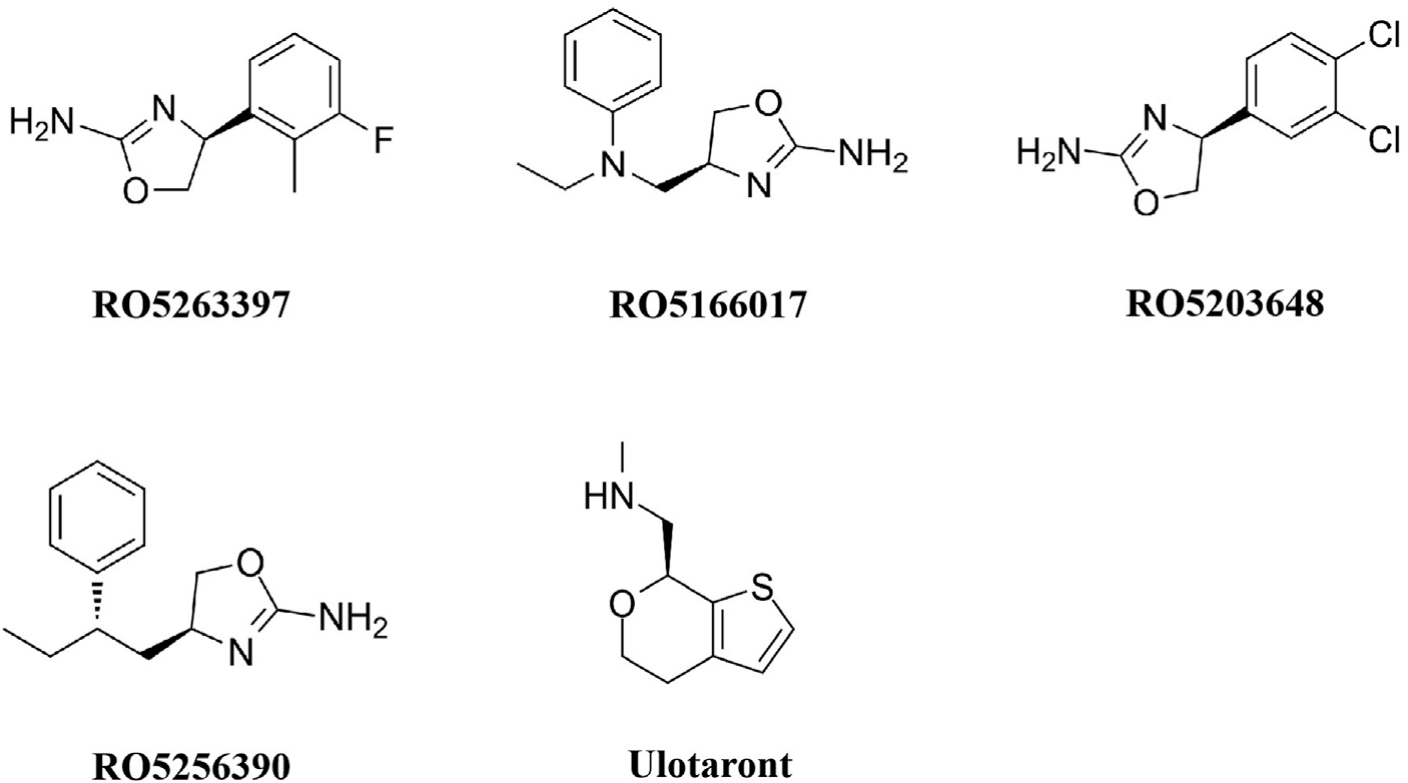
Structural formulae of TAAR1 agonists tested in stress-related disorders.

As mentioned above, the roles of DA, 5-HT, and NE in mental disorders have gained significant attention from researchers. Consequently, the understanding of trace amines has been largely overlooked. Several years after the discovery of TAARs, a group of compounds that selectively activate TAAR1 were engineered. These compounds include partial TAAR1 agonists, such as RO5263397, and full TAAR1 agonists, such as RO5166017 and RO5203648 (Figure 1). Recent evidence over the past two decades suggests that TAAR1 agonists can influence stress-related behaviors and may serve as potential pharmacotherapeutics for stress-related disorders. These findings have significantly enhanced our understanding of the trace amine system in relation to stress responses and associated mental disorders.

## TAAR1 agonists and major depressive disorder

Several TAAR1 agonists have been evaluated in preclinical models of depression, showing potential antidepressant-like properties (Table 1). The TAAR1 partial agonist RO5203648 significantly reduced immobility of rats in the forced swimming test (FST), a behavioral assessment sensitive to the effects of antidepressants (Revel et al., 2012). Similarly, the TAAR1 partial agonist RO5263397 decreased immobility time in the FST in rats (Revel et al., 2013). In contrast, the full agonist RO5256390 did not affect this paradigm (Revel et al., 2013). In a differential reinforcement of low-rate behavior study conducted with monkeys, RO5203648 showed a trend toward an increased number of reinforcers obtained, which also led to a significant prolongation of the inter-response time and a reduction in the response rate, effects similar to those of antidepressants (Revel et al., 2012; O'Donnell et al., 2005). Likewise, both RO5263397 and RO5256390 increased the number of reinforcers obtained in this study (Revel et al., 2013). In addition, RO5263397 was effective in reversing social avoidance behaviors and cognitive impairments induced by chronic social defeat stress (CSDS) in mice, a widely used model of depression (Zhang et al., 2021). Notably, preclinical studies have highlighted the potential of ulotaront (SEP-363856), a TAAR1 agonist currently in II/III clinical trials to treat schizoprenia, MDD, and anxiety. A study assessed the antidepressant effects of ulotaront across various doses (0.1–10 mg/kg, p.o.) using both FST and tail suspension tests (TST) in mice, revealing that ulotaront significantly reduced immobility in these tests, suggesting its potential antidepressant effects (Ren et al., 2022; Dedic et al., 2019). A more recent study confirmed that ulotaront (10 mg/kg, p.o.) could prevent the reductions in sucrose preference and molecular changes in the hippocampus induced by chronic unpredictable mild stress (CUMS) in mice, suggesting the antidepressant-like properties of ulotaront (Li et al., 2024). However, it is crucial to note that even at a dose of 10 mg/kg (p.o.), ulotaront may induce general inhibition of locomotion and body temperature in C57BL/6J mice, an effect that was mediated by TAAR1 (Saarinen et al., 2022). Due to the significant interference of general inhibition in behavioral tests and potential side effects in clinical applications, future research should focus more on the dose-dependent effects of ulotaront. In addition, previous studies have proposed that TAAR1 agonists may produce both TAAR1-dependent and -independent effects on depressive-like behaviors in mice (Mantas et al., 2021). While ulotaront activates both TAAR1 and 5-HT1A receptors, it remains unclear whether the antidepressant-like effects are primarily mediated by TAAR1.

**TABLE 1:** Summary of the effects of TAAR1 agonists on stress-related disorders in animals.

TAAR1 agonists	Psychiatric disorders	Behaviors	Effects	References
RO5166017	Anxiety	Stress-induced hyperthermia	Attenuated	Revel et al. (2011)
	PTSD	Stress-enhanced fear learning	Attenuated	Peng et al. (2024)
RO5256390	Anxiety	Elevated plus maze Open field test	No change Decreased time spent in central zone	Raony et al. (2022)
	Depression	Forced swim test A differential reinforcement for low-rate paradigm	No change Increased the number of reinforcers obtained but decreased the response rate	Revel et al. (2013)
RO5263397	Anxiety	Stress-induced hyperthermia Nicotine withdrawal-induced anxiety	Attenuated Attenuated	Revel et al. (2011), Zhang et al. (2021), Wu et al. (2022)
		Elevated plus maze	No change	
		Chronic stress-induced anxiety	No change	
	PTSD	Stress-enhanced fear learning Single prolonged stress-induced extinction impairment	Attenuated Attenuated extinction impairment	Peng et al. (2024)
	Depression	Forced swim test A differential reinforcement for low-rate paradigm Chronic stress-induced social avoidance	Attenuated immorbility Increased the number of reinforcers obtained but decreased the response rate Reversed	Revel et al. (2013), Zhang et al. (2021), Zhang et al. (2024)
RO5203648	Anxiety	Stress-induced hyperthermia	Attenuated	Revel et al. (2011)
	Depression	Forced swim test Tail suspension test A differential reinforcement for low-rate paradigm	Attenuated immorbility Attenuated immorbility Prolonged the inter-response time and a reduction in the response rate	Revel et al. (2012)
SEP363856	Depression	Forced swim test Tail suspension test Chronic unpredicted mild stress (CUMS)	Attenuated immorbility Attenuated immorbility Attenuated CUMS-induced reductions in sucrose preference	Ren et al. (2022), Dedic et al. (2019), Li et al. (2024)
PCC0105004	Bipolar disorders	Ouabain-induced biplolar-like behaviors	Attenuated	Yu et al. (2024)

Some studies have indicated the potential neural mechanisms related to trace amines and TAAR1 in depression. For example, consistent with the observed antidepressant-like properties, RO5263397 mitigated CSDS-induced morphological alterations in neurons within the medial prefrontal cortex (mPFC) and the dentate gyrus (DG) subregion of the hippocampus. Additionally, genetic intervention targeting TAAR1 in the mPFC and DG could modulate CSDS-induced social avoidance and cognitive impairment in mice (Zhang et al., 2021; Zhang et al., 2024). These findings indicate that TAAR1 in the mPFC and DG is involved in mediating chronic stress-induced depressive-like behaviors. It is also important to recognize that various trace amines are metabolized by monoamine oxidase (MAO), which is the main target of clinically used antidepressants known as monoamine oxidase inhibitors (MAOIs) (Shulman et al., 2013). In clinical settings, MAO inhibitors such as tranylcypromine (TCP) can effectively alleviate symptoms of depression but may also cause side effects related to elevated tyramine levels in the body. A recent study found that TCP led to higher accumulation of the trace amine tyramine in the striatum of TAAR1 knockout (KO) mice, indicating a negative feedback mechanism by TAAR1 in regulating tyramine levels (Mantas et al., 2021). Interestingly, TCP produced stronger antidepressant-like effects in TAAR1-KO animals compared to wild-type mice (Mantas et al., 2021). Both TCP and tyramine were shown to reduce glutamate release in the substantia nigra of wild-type mice, but this effect was not observed in TAAR1-KO mice, suggesting that TAAR1 mediates TCP-induced hypoglutamate transmission (Mantas et al., 2021). These findings reveal a complex role of tyramine and the regulation of TAAR1 in the antidepressant properties of MAOIs, highlighting the need for further exploration of the contributions of the trace amine system to MAOIs. Despite concerns about side effects among clinicians and a lack of industry support, MAOIs continue to be effective antidepressants widely used in clinical practice and warrant further investigation, especially in light of the recent resurgence of research on the trace amine system (Shulman et al., 2013).

## TAARs and bipolar disorders

Compared to the widely accepted notion that trace amine levels are deficient in cases of depression, the levels of trace amines in individuals with bipolar disorders remain less clearly defined. Early studies that explored the relationship between depressive disorders and trace amine levels often included patients diagnosed with bipolar disorder, which complicates the clarity of the findings (Sandler et al., 1979; Sabelli and Mosnaim, 1974). In patients experiencing depressive symptoms related to bipolar disorder, it has been shown a decrease in the urinary excretion of β-PEA, suggesting that lower levels of β-PEA may correlate with depressive states of bipolar disorder (Sandler et al., 1979; Sabelli and Mosnaim, 1974). Conversely, during manic episodes, studies have indicated an increase in β-PEA levels (Fischer et al., 1972). A preliminary report further indicated that certain female patients diagnosed with primary major bipolar affective disorders exhibited a significantly higher mean excretion rate of β-PEA than expected (Karoum et al., 1982). Collectively, these findings suggest a potential link between trace amines and bipolar disorder.

In addition to these biochemical observations, genetic analyses have begun to illuminate the possible role of TAARs in the etiology of bipolar disorder. A family-based association study pointed towards a potential involvement of the TAAR6 locus in the development of bipolar disorder, while the loci for TAAR1 and TAAR5 showed no significant associated risk (Abou Jamra et al., 2005). However, these findings were not replicated in a more extensive subsequent study, which raises questions regarding their reliability (Liu et al., 2007; Venken et al., 2006). Furthermore, it has been suggested that certain genetic variants of TAAR1 may be present in some bipolar patients, although this association requires deeper exploration and validation (Rutigliano et al., 2019). Beyond the genetic factors, animal studies have provided compelling evidence for the involvement of TAAR1 in the pathophysiology of bipolar disorder. For instance, it has been demonstrated that TAAR1 can activate both Gαs and βArr2 signaling pathways in rats. These signaling cascades are particularly affected by the interaction between TAAR1 and dopamine D2 receptors (D2R) (Harmeier et al., 2015). When D2R are present, the activation of TAAR1 appears to recruit βArr2 signaling pathways, leading to an increased phosphorylation of Akt and a marked inhibition of GSK3β phosphorylation. This finding is particularly interesting considering that GSK3 inhibitors, like lithium, have been widely recognized as one of the most effective treatments for managing bipolar disorder (Malhi and Outhred, 2016; Fountoulakis et al., 2022).

Given the role of TAAR1 in GSK3β regulation, it is reasonable to hypothesize that TAAR1 agonists may influence the progression of bipolar disorder by modulating GSK3β signaling pathways. In an animal model of bipolar disorder induced by ouabain, PCC0105004, a novel TAAR1 agonist, was shown to produce potential antimanic-like and antidepressant-like efficacy in mice. Molecular data revealed that PCC0105004 was able to reverse the alterations in Akt/GSK3β signaling induced by ouabain (Yu et al., 2024). In line with this, our unpublished observations showed that pharmacological activation of TAAR1 using other agonists could similarly be effective in altering bipolar-related behaviors in rats. Since the effects of TAAR1 agonists on bipolar disorder may arise from biased regulation of GSK3β pathways, it would be insightful to investigate whether biased TAAR1 agonists could yield different behavioral outcomes in subsequent studies. Although these findings are intriguing, our understanding of TAAR1 and its specific role in bipolar disorder remains largely unclear.

## TAARs in general anxiety and post-traumatic stress disorder

Research has examined the potential anxiolytic effects of selective TAAR1 agonists on various anxiety-like behaviors. One compelling model used to evaluate these effects is the stress-induced hyperthermia paradigm, which measures increases in body temperature as a physiological response to mild stress. In this context, both the TAAR1 full agonist RO5166017 and the partial agonist RO5203648 have shown a significant ability to prevent acute stress-induced hyperthermia, suggesting that these compounds may possess anxiolytic-like properties in mice (Revel et al., 2011; Revel et al., 2012). Furthermore, the anxiolytic effects of RO5166017 were not observed in *taar1-*KO mice, providing strong evidence that these effects depend on the presence of TAAR1 (Revel et al., 2011). Additionally, a study revealed that the TAAR1 partial agonist RO5263397 induced significant anxiolytic effects in animals undergoing withdrawal from prolonged nicotine self-administration but not in pharmacologically naive rats (Wu et al., 2022). Our recent research explored the effects of both RO5166017 and RO5263397 in animal models of post-traumatic stress disorder (PTSD): single prolonged stress-induced impairment of fear extinction and stress-enhanced fear learning, and indicated that RO5263397 and RO5166017 could attenuate PTSD-like behaviors in rats (Peng et al., 2024).

However, there are also controversial findings on the anxiolytic effects of TAAR1 agonists. For instance, one study demonstrated that RO5263397 did not alter CSDS-induced anxiety-like behaviors, as evaluated through the elevated plus maze and open field (OF) tests in mice (Zhang et al., 2021). Additionally, the TAAR1 full agonist RO5256390 did not significantly affect the percentage of entries into the open arms of the elevated plus maze (EPM) test. In contrast, the TAAR1 antagonist EPPTB increased these entries, suggesting a potential anxiolytic property for EPPTB in rats (Raony et al., 2022). It should be noted that, this study was conducted using a single concentration of these compounds, and their pharmacological effects may be dose-dependent, which requires further investigation in the future. Interestingly, RO5256390 led to a reduction in central distance in the OF test, indicating possible anxiogenic properties in rats (Raony et al., 2022). These findings highlight the effects of different TAAR1 agonists may vary based on the specific compound and the physiological state of the animals involved. Overall, the literature supports the idea that TAAR1 agonists could serve as promising pharmacological treatments for anxiety-related disorders. One notable development in this field is the evaluation of ulotaront, a dual agonist for TAAR1 and the 5HT1A receptor, in a Phase 2/3 clinical trial aimed at treating generalized anxiety disorder.

In addition to TAAR1, the emerging role of the TAAR5 receptor in anxiety-like behaviors is an intriguing area of research. Studies have shown that genetic deletion of the *taar5* gene results in reduced anxiety-like behaviors across various anxiety behavioral assessments in mice (Espinoza et al., 2020). This deletion has also been associated with alterations in monoaminergic transmission within brain regions such as the striatum and hypothalamus, emphasizing the potential impact of TAAR5 in regulating psychiatric disorders (Espinoza et al., 2020; Efimova et al., 2021). Our unpublished observations align with these findings, suggesting that a putative non-selective TAAR5 agonist, alpha-NETA, exhibits anxiogenic properties in rats. Previous studies have indicated that alpha-NETA can induce psychotic-like behavioral abnormalities and affect dopamine transmission in the striatum, revealing that TAAR5 might participate in psychiatric disorders in regulating monoaminergic system (Belov et al., 2020). Unlike TAAR1, which is not expressed in the olfactory bulb, TAAR5 is prominently expressed in the glomerular layer of the olfactory bulb in mice. Moreover, TAAR5 is found in deeper layers projecting to the limbic olfactory circuitry and in various limbic brain regions, such as the amygdala, hippocampus, and hypothalamic nuclei, which are critical areas involved in the stress response and anxiety regulation (Espinoza et al., 2020). Future studies are needed to develop potential TAAR5 antagonists for anxiety treatment and to further explore the neural mechanisms through which TAAR5 regulates anxiety.

## Conclusion

While the trace amine system’s involvement in psychiatric disorders like schizophrenia and drug addiction has garnered significant attention in research, the role of TAAR1 in stress response and related afflictions has not been as thoroughly explored. In addition, current studies of TAAR1 in animals have largely focused on males, with females being significantly overlooked. Emerging evidence suggests sex is an important factor influencing neuropharmacological responses, highlighting an important consideration for future research on the pharmacological properties of TAAR1 ligands. Nonetheless, a growing body of evidence indicates that TAAR1 is crucial in modulating responses to stress and may contribute to the development of stress-related disorders. In addition, TAAR5 has emerged as a potential regulator of anxiety, suggesting that these trace amine receptors could play key roles in mental health. Traditional antidepressants and anxiolytics, including serotonin reuptake inhibitors, serotonin-norepinephrine reuptake inhibitors, and benzodiazepines, are effective treatments in clinical practice. However, these medications are frequently accompanied by undesirable side effects, such as cognitive decline, weight gain, obesity, sleep disturbances, and even diabetes. In contrast, preliminary studies on TAAR1 agonists reveal a promising ability to enhance cognitive function without leading to catalepsy or weight gain, indicating a potentially safer alternative for patients (Liu et al., 2024; Revel et al., 2013; Black et al., 2017; Zhang et al., 2021).

Despite these insights, our understanding of the underlying mechanisms through which TAAR1 affects stress-related disorders remains largely insufficient. TAAR1 is expressed in several critical brain regions, including the prefrontal cortex, hippocampus, terminal bed nucleus, lateral tegmental area, and ventral raphe nucleus, which have been widely implicated in stress response, emotional process, and mood regulation. To further unravel the complex underpinnings of TAAR1’s function in the brain, future studies are necessary to identify the specific regions where TAAR1 exerts its influence in regulating stress-related conditions. Current evidence strongly suggests that TAAR1 agonists could represent potential pharmacotherapy options for managing stress-related disorders, including depression, bipolar disorder, and anxiety disorders such as PTSD, offering hope for more effective and safer treatments in the realm of mental health.
